# Premorbid Metabolic Syndrome Is Associated with the Hypoinflammatory Phenotype in Acute Respiratory Distress Syndrome and Sepsis

**DOI:** 10.1164/rccm.202503-0596RL

**Published:** 2025-10-10

**Authors:** Avery M. Bogart, Sarah N. Obeidalla, Rombout B. E. van Amstel, Olaf Cremer, Lieuwe D. J. Bos, Brian Bartek, Pratik S. Sinha, Carolyn S. Calfee, V. Eric Kerchberger, Lorraine B. Ware, the MARS Consortium, Friso M. de Beer

**Affiliations:** ^1^ Department of Pathology, Microbiology, and Immunology,; ^2^ Department of Biomedical Informatics, and; ^3^ Division of Allergy, Pulmonary, and Critical Care Medicine, Department of Medicine, Vanderbilt University Medical Center, Nashville, Tennessee;; ^4^ Vanderbilt University School of Medicine, Nashville, Tennessee;; ^5^ Department of Intensive Care Medicine, Amsterdam University Medical Center, Locatie Amsterdam Medical Center, Amsterdam, the Netherlands;; ^6^ Department of Intensive Care, University Medical Center Utrecht, Utrecht, the Netherlands;; ^7^ Division of Clinical and Translational Research, Department of Anesthesia, Washington University in St Louis, St. Louis, Missouri; and; ^8^ Division of Pulmonary, Critical Care, Allergy, and Sleep Medicine, Department of Medicine and; ^9^ Department of Anesthesia, University of California, San Francisco, San Francisco, California

*To the Editor*:

Sepsis and acute respiratory distress syndrome (ARDS) pose a substantial burden to public health (1). Recent work identified two phenotypes, hyper- and hypoinflammatory, in ARDS and sepsis (2, 3). Patients with the hyperinflammatory phenotype have greater illness severity and mortality, as well as differential responses to treatment (2–4). However, the mechanisms driving the separation between these phenotypes is unclear. Our study sought to provide insight into the heterogeneity of host responses in critical illness by defining if and how patient-specific factors associate with either phenotype. Preexisting medical conditions such as metabolic syndrome (MetS) could contribute to critical illness phenotypes by influencing the inflammatory response to infection. MetS is characterized by a chronic, low-grade inflammatory state, which could modify the immune response to an acute insult (5). A recent study of adults with coronavirus disease (COVID-19) found that MetS was associated with increased risks for ARDS, severe illness, and death (5). We also previously reported that chronic hyperglycemia, a feature of MetS, is associated with increased illness severity and the risk for ARDS in patients with sepsis (6). Given that critically ill patients with the hyperinflammatory phenotype similarly have worse clinical outcomes, we hypothesized that premorbid MetS would increase the risk for this phenotype among patients with ARDS or sepsis. Some of these results were previously reported in abstracts (7, 8).

## Methods

We studied 3,142 patients with sepsis or ARDS from three prospective cohorts of critically ill adults: VALID (Validation of Acute Lung Injury biomarkers for Diagnosis; enrollment, 2007–2016; Nashville, TN), EARLI (Early Assessment of Renal and Lung Injury; enrollment, 2008–2019; San Francisco, CA), and MARS (Molecular Diagnosis and Risk Stratification of Sepsis; enrollment, 2011–2014; Amsterdam, The Netherlands). These cohorts represent large clinically and demographically diverse populations, are well phenotyped for ARDS and sepsis, and have a variety of ARDS risk factors. Data were missing at random. MetS was defined per the modified World Health Organization criteria (5). Because of the low prevalence of MetS in each cohort, the data were combined for analysis. Component MetS diagnoses (hypertension, diabetes, obesity, dyslipidemia) were collected from the electronic medical record at enrollment. In EARLI, diagnosis of dyslipidemia was not recorded; EARLI patients were defined as having MetS when the other three diagnoses were present. Phenotype assignment was derived by latent class analysis using the same clinical and biomarker data as previously (2–4). A probability cutoff of ⩾0.5 was used to assign the hyperinflammatory phenotype (4). The association between MetS or its component diagnoses and phenotypes was analyzed in univariable and multivariable analyses controlling for age, sex, and Sequential Organ Failure Assessment score. These covariates were selected to account for differences in demographic characteristics and illness severity between the geographically distinct cohorts. A mixed-effects model with random intercepts was employed to assess the impact of study site on the relationship between MetS and phenotype.

## Results

**Figure 1.**
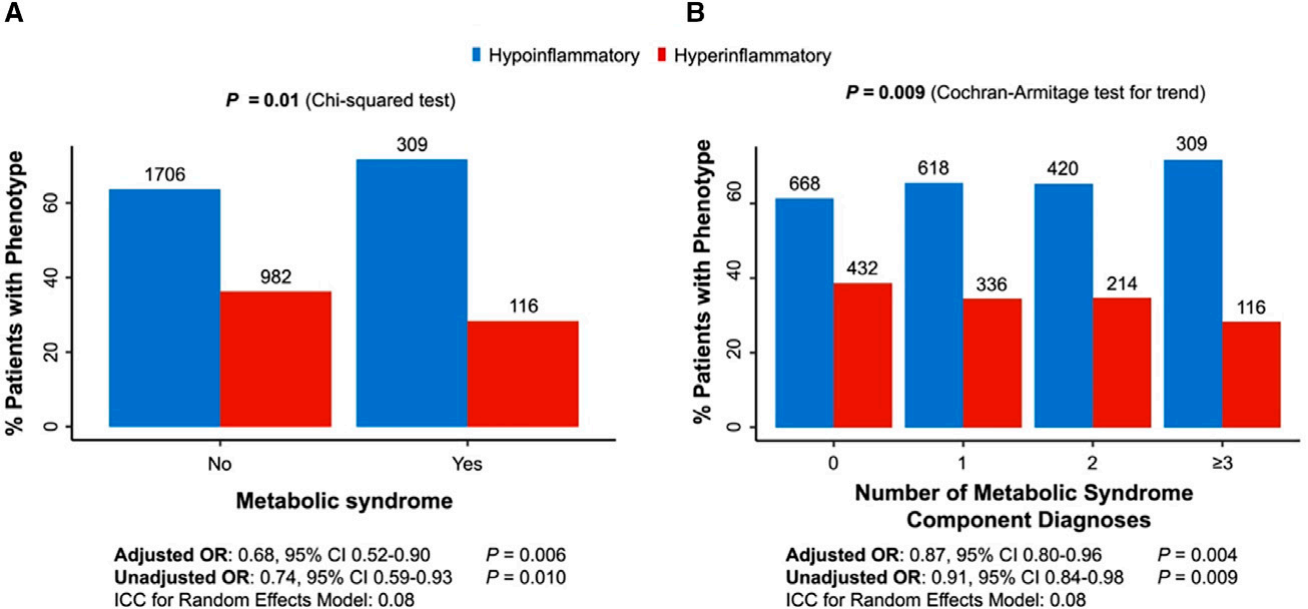
Association of premorbid (*A*) metabolic syndrome (MetS) and (*B*) number of component diagnoses of MetS with inflammatory phenotypes in 3,142 patients with acute respiratory distress syndrome and sepsis. (*A*) Bar plot depicting the percentage of patients with each phenotype stratified by the presence of MetS. Comparisons were made by univariable analysis (χ^2^ test, results reported above the graph) and multivariable logistic regression modeling with random intercepts (results reported below the graph). The adjusted model controlled for age, sex, and Sequential Organ Failure Assessment score. (*B*) Bar plot depicting the percent of patients with each phenotype stratified by number of component diagnoses for MetS. Comparisons were made by univariable analysis (Cochran-Armitage test for trend, results reported above the graph) and multivariable logistic regression modeling with random intercepts (results reported below the graph). The adjusted model controlled for age, sex, and Sequential Organ Failure Assessment score. ICC = intraclass correlation coefficient; OR = odds ratio.

Among the 3,142 patients, 2,036 (64.80%) were classified as having hypoinflammatory disease and 1,106 (35.20%) were classified as having hyperinflammatory disease. Demographic and clinical data for each cohort are shown in Table 1. Unexpectedly, premorbid MetS was associated with the hypoinflammatory phenotype (*P* = 0.01; Figure 1A), and the proportion of patients classified as having hyperinflammatory disease decreased with each additional MetS component diagnosis (*P* = 0.009; Figure 1B). The association between MetS and lower odds of the hyperinflammatory classification persisted when controlling for age, sex, and Sequential Organ Failure Assessment score (odds ratio for hyperinflammatory phenotype, 0.68; 95% confidence interval, 0.52–0.90; Figure 1A). The intraclass correlation coefficient (ICC), which quantifies the proportion of variance attributable to differences between study sites, was 0.08. This modest ICC indicates that, although most of the variability is at the individual level, a nonnegligible proportion of effect may be explained by site-level clustering. Additionally, having more component diagnoses for MetS was associated with decreased odds of being classified as having the hyperinflammatory phenotype in a logistic regression analysis controlling for the same covariates (odds ratio of hyperinflammatory phenotype for each additional MetS component diagnosis, 0.87; 95% confidence interval, 0.80–0.96; ICC, 0.08; Figure 1B). Hospital mortality rates were also compared between phenotypes, stratified by MetS. Among patients with the hypoinflammatory phenotype, mortality was lower in patients with MetS compared with those without MetS (16% vs. 21%; *P* = 0.021). By contrast, in patients with the hyperinflammatory phenotype, there were no differences in mortality between those with and without MetS (48% vs. 44%; *P* = 0.5).

**Table 1.** Comparison of Demographic and Clinical Data between VALID, EARLI, and MARS

Characteristic	VALID (*N* = 1,134)	EARLI (*N* = 870)	MARS (*N* = 1,138)	*P* Value
Female sex	513 (45%)	380 (44%)	489 (43%)	0.5
Age, yr				
Median	58	65	62	<0.001
IQR	47–67	65–75	51–71	
White race	968 (85%)	420 (48%)	—	—
Metabolic syndrome	269 (24%)	43 (4.9%)	126 (11%)	<0.001
Component diagnoses for MetS				
0	275 (24%)	341 (39%)	528 (46%)	<0.001
1	314 (28%)	318 (37%)	298 (26%)	
2	276 (24%)	168 (19%)	186 (16%)	
3	185 (16%)	43 (4.9%)	101 (8.9%)	
4	84 (7.4%)	—	25 (2.3%)	
Obesity	374 (37%)	173 (22%)	200 (18%)	<0.001
Diabetes	366 (32%)	237 (27%)	228 (20%)	<0.001
Hypertension	650 (57%)	373 (43%)	327 (29%)	<0.001
Hyperlipidemia	367 (32%)	—	318 (28%)	—
Hyperinflammatory LCA subphenotype	336 (40%)	304 (35%)	466 (41%)	<0.001

*Definition of abbreviations*: EARLI = Early Assessment of Renal and Lung Injury; LCA = latent class analysis; MARS = Molecular Diagnosis and Risk Stratification of Sepsis; MetS = metabolic syndrome; VALID = Validation of Acute Lung Injury biomarkers for Diagnosis.

Hyperlipidemia data were not recorded in the EARLI cohort. Race and ethnicity information was not available in the MARS cohort because of European restrictions on data collection and distribution.

## Discussion

Contrary to our hypothesis, premorbid MetS increased the likelihood of assignment to the hypoinflammatory rather than the hyperinflammatory phenotype in patients with sepsis or ARDS. The significance of this finding is reinforced by the lower mortality rate in patients with hypoinflammatory disease, which appears to be even lower in those with MetS as demonstrated in our study (2, 4). This finding is consistent with previous studies investigating the relationship between MetS and mortality in non–COVID-19 ARDS (9). The etiology of this association is uncertain but raises the possibility of immunomodulatory effects from MetS that alter the inflammatory trajectory in acute illness. This is supported by the obesity paradox, whereby patients with obesity are more likely to develop ARDS but have better outcomes than nonobese patients, although this remains a topic of considerable debate (10, 11). Additionally, it has been reported that having obesity or diabetes is associated with impaired vaccine responses (12). Further, mitochondrial dysfunction and epigenetic reprogramming are both features of MetS and could lead to reduced immune responses to acute insults (13, 14). Alternatively, different infectious etiologies and medications used to treat MetS component diagnoses may impact immune function, representing important potential confounders in this study; microbiologic data and prehospital medication history were not uniformly available across the study cohorts for analysis. Additionally, we cannot exclude the possibility of collider bias, whereby hospitalization and intensive care unit admission may be conditioning factors influencing our results. However, our findings do not imply a causal effect of MetS but rather support future hypothesis-driven studies by offering insight into how preexisting comorbidities may influence molecular phenotypes in critical illness. Subsequent investigations will be important to determine the basis for the association between MetS and the hypoinflammatory phenotype in critical illness.
